# Supplementary material for: Bacterial Diversity and Population Dynamics During the Fermentation of Palm Wine From Guerrero Mexico
Source: Front Microbiol. 2019 Mar 22;10:531. doi: 10.3389/fmicb.2019.00531 (PMC6440455; doi:10.3389/fmicb.2019.00531)
Supplement: Supplementary file 1 [file Data_Sheet_1.docx]

Supplementary material.

Figures.


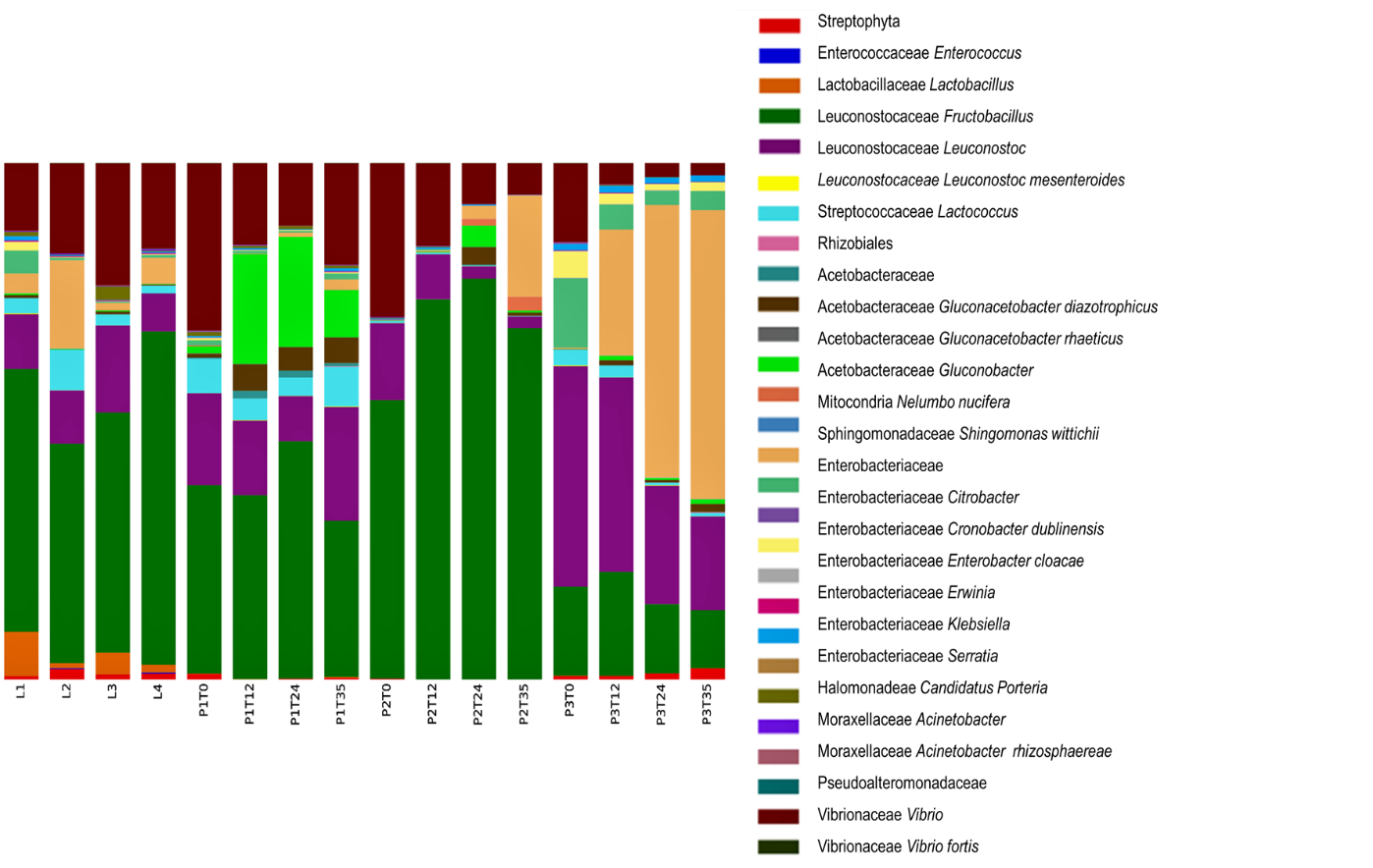


**Figure 1S. Taxonomic identification.** Most abundant OTU’s using the 0.01% abundance filter OTUs table.


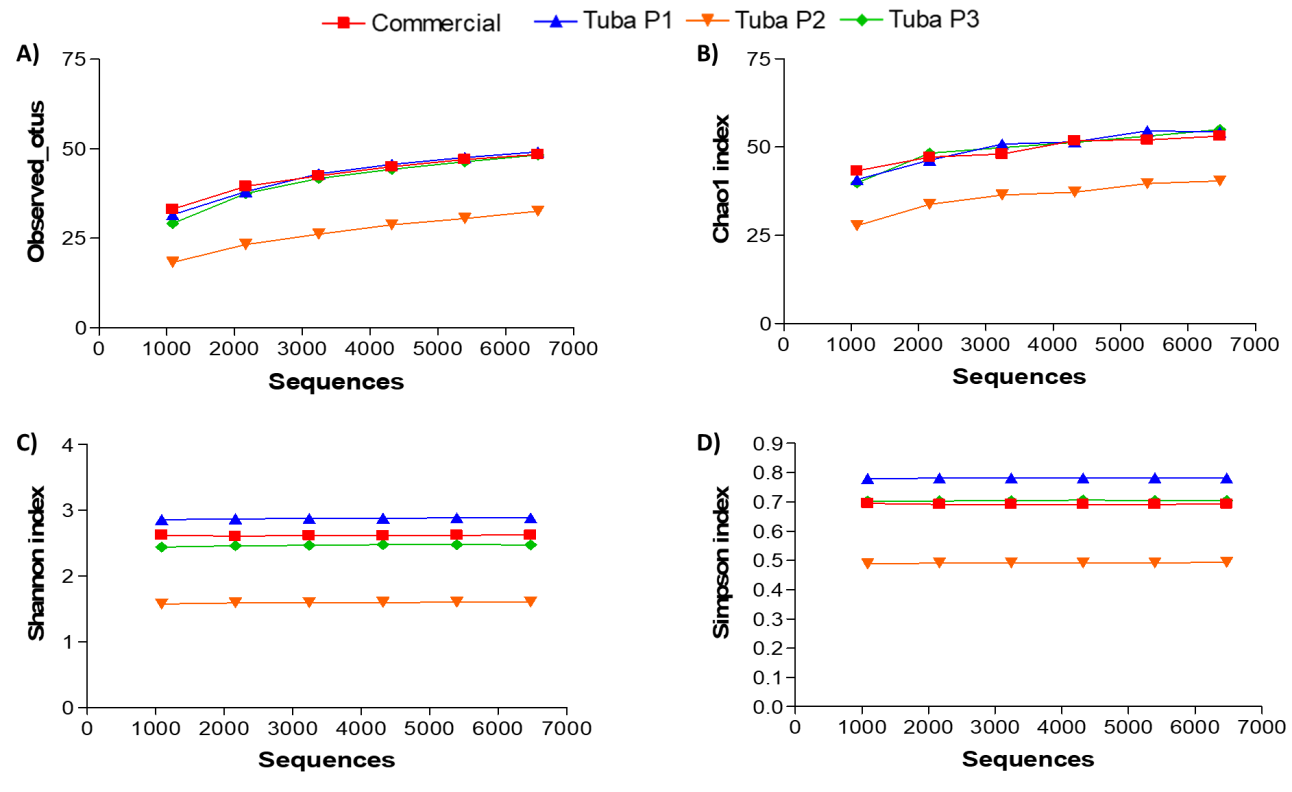


**Figure 2S. Alpha diversity rarefaction plots with 0.01%.** A) Observed_otus, B) Chao1, C) Shannon and D) Simpson. Each population is represented for a specific color in all the graphics**.**


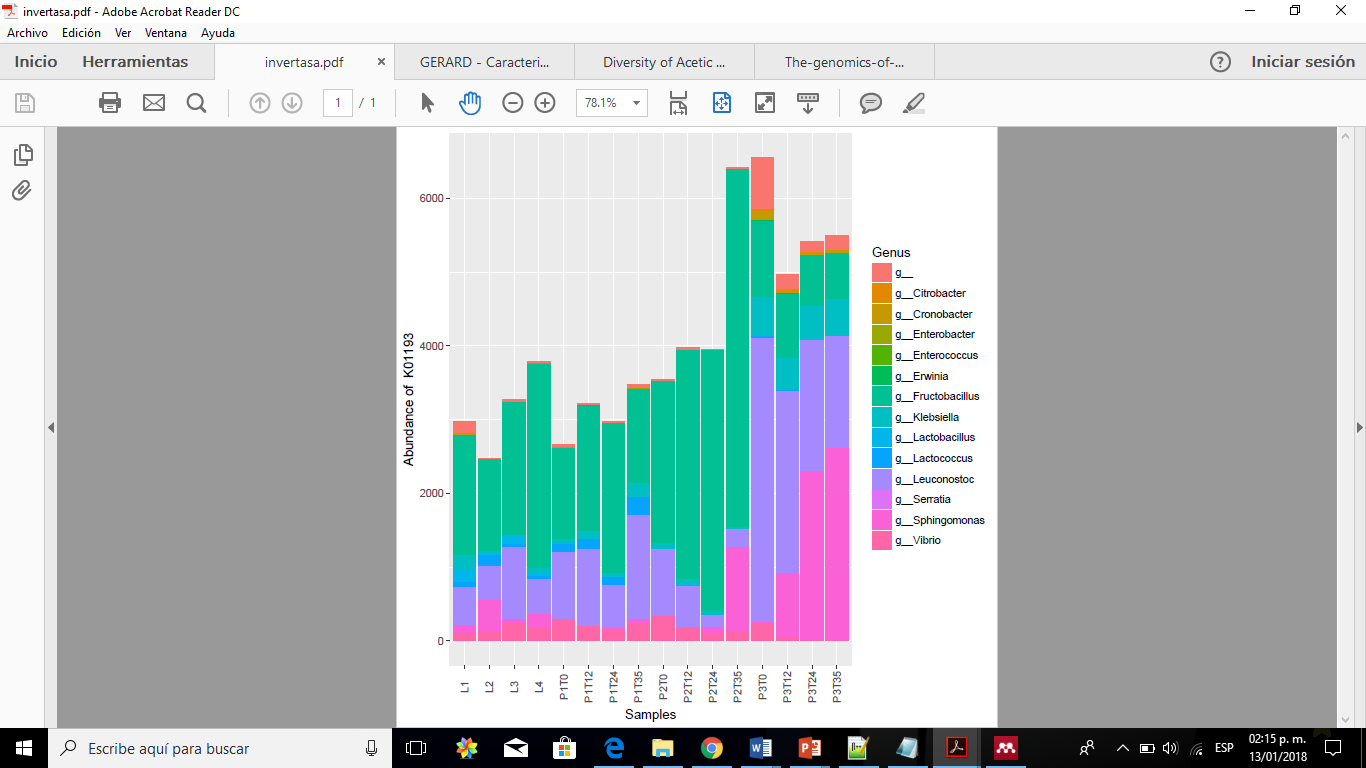


**Figure 3S. Abundance of invertase gene (K01193).** Analysis performed with the function “metagenome_contributions.py” obtained by PICRUSt analysis and plotted with R studio.


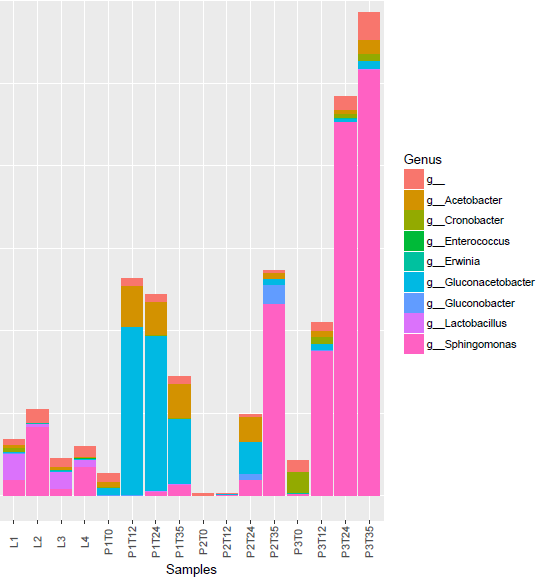


**Figure 4S. Main bacteria with 15-cis-phytoene synthase gene (K02291 KEGG code).** Analysis performed with the function “metagenome_contributions.py” obtained by PICRUSt analysis and plotted with R studio.

**Tables.**

**Table 1S. Chemical composition of the Tuba.**

|  | **Tuba P1** | **Tuba P2** | **Tuba P3** | **Commercial L1** | **Commercial L2** | **Commercial L3** | **Commercial L4** |
| --- | --- | --- | --- | --- | --- | --- | --- |
| **Time (h)** | **Proteins (g/L)** | | | | | | |
| 0 | 0.068 | 0.115 | 0.097 | 0.087 | 0.173 | 0.166 | 0.054 |
| 12 | 0.094 | 0.038 | 0.109 |  |  |  |  |
| 24 | 0.057 | 0.084 | 0.046 |  |  |  |  |
| 35 | 0.082 | 0.147 | 0.042 |  |  |  |  |
| **Time (h)** | **Sucrose (g/L)** | | | | | | |
| 0 | 85.147 | 121.760 | 95.203 | 45.310 | 13.455 | 99.291 | 4.316 |
| 12 | 0.393 | 113.293 | 19.837 |  |  |  |  |
| 24 | 0.374 | 102.178 | 0.883 |  |  |  |  |
| 35 | 0.262 | 4.206 | 0.747 |  |  |  |  |
| **Time (h)** | **Glucose (g/L)** | | | | | | |
| 0 | 21.146 | 13.179 | 14.665 | 59.240 | 39.814 | 35.078 | 51.846 |
| 12 | 61.414 | 3.452 | 39.814 |  |  |  |  |
| 24 | 60.144 | 21.649 | 7.264 |  |  |  |  |
| 35 | 21.036 | 59.050 | 0.887 |  |  |  |  |
| **Time (h)** | **Fructose (g/L)** | | | | | | |
| 0 | 16.016 | 11.078 | 11.922 | 50.874 | 45.528 | 32.765 | 40.953 |
| 12 | 47.358 | 1.918 | 29.177 |  |  |  |  |
| 24 | 48.562 | 13.028 | 39.734 |  |  |  |  |
| 35 | 44.836 | 45.633 | 28.122 |  |  |  |  |
| **Time (h)** | **Total sugars (g/L)** | | | | | | |
| 0 | 122.311 | 146.018 | 121.791 | 155.425 | 98.798 | 167.135 | 97.116 |
| 12 | 109.166 | 118.665 | 88.829 |  |  |  |  |
| 24 | 109.081 | 136.856 | 47.882 |  |  |  |  |
| 35 | 66.135 | 108.890 | 29.757 |  |  |  |  |
| **Time (h)** | **Acetate (g/L)** | | | | | | |
| 0 | 0.686 | 0.893 | 0.645 | 1.520 | 1.644 | 1.527 | 1.984 |
| 12 | 3.067 | 1.406 | 1.104 |  |  |  |  |
| 24 | 6.914 | 2.764 | 2.835 |  |  |  |  |
| 35 | 6.074 | 3.559 | 3.957 |  |  |  |  |
| **Time (h)** | **Ethanol (g/L)** | | | | | | |
| 0 | 0.411 | 0.166 | 0.252 | 1.517 | 1.419 | 0.701 | 4.420 |
| 12 | 2.239 | 0.000 | 1.175 |  |  |  |  |
| 24 | 4.427 | 0.000 | 41.365 |  |  |  |  |
| 35 | 47.114 | 6.806 | 50.692 |  |  |  |  |
| **Time (h)** | **pH** | | | | | | |
| 0 | 3.73 | 3.65 | 3.64 | 4.0 | 4.0 | 4.0 | 4.0 |
| 4 | 3.34 | 3.38 | 3.3 |  |  |  |  |
| 10 | 3.15 | 3.21 | 3.12 |  |  |  |  |
| 12 | 3.1 | 3.19 | 3.11 |  |  |  |  |
| 24 | 2.93 | 3.12 | 2.98 |  |  |  |  |
| 35 | 2.94 | 2.81 | 2.94 |  |  |  |  |
